# Femtosecond infrared spectroscopy of channelrhodopsin-1 chromophore isomerization

**DOI:** 10.1063/1.4948338

**Published:** 2016-04-29

**Authors:** T. Stensitzki, Y. Yang, V. Muders, R. Schlesinger, J. Heberle, K. Heyne

**Affiliations:** 1 Department of Physics, Institute of Experimental Physics, Freie Universität Berlin, Arnimallee 14, 14195 Berlin, Germany; 2 Genetic Biophysics, Department of Physics, Freie Universität Berlin, Arnimallee 14, 14195 Berlin, Germany; 3 Experimental Molecular Biophysics, Department of Physics, Freie Universität Berlin, Arnimallee 14, 14195 Berlin, Germany

## Abstract

Vibrational dynamics of the retinal all-*trans* to 13-*cis* photoisomerization in channelrhodopsin-1 from *Chlamydomonas augustae* (*Ca*ChR1) was investigated by femtosecond visible pump mid-IR probe spectroscopy. After photoexcitation, the transient infrared absorption of C-C stretching modes was detected. The formation of the 13-*cis* photoproduct marker band at 1193 cm^−1^ was observed within the time resolution of 0.3 ps. We estimated the photoisomerization yield to (60 ± 6) %. We found additional time constants of (0.55 ± 0.05) ps and (6 ± 1) ps, assigned to cooling, and cooling processes with a back-reaction pathway. An additional bleaching band demonstrates the ground-state heterogeneity of retinal.

## I. INTRODUCTION

Light excitation of rhodopsins lead to various functionalities like sensing, ion pumping and channeling across the biological membrane. Channelrhodopsins (ChR) are the only light-gated ion channels in nature found so far. Originally, they are located in the eyespot of green algae to mediate phototaxis. In these days, channelrhodopsins are used in the vibrant field of optogenetics^1^ where the protein is used to elicit action potentials in nerve cells by light. ChR have been applied to unravel neuronal connectivity^2^ and to manipulate behavior in ChR-expressing animals like worms and rodents.^3,4^ Due to their application in living organisms, the detailed understanding of the molecular mechanism after light excitation is of high interest.

Common to all rhodopsins, the initial step of photo-activation of channelrhodopsin involves isomerization of the retinal chromophore. Most spectroscopic analysis has been performed on channelrhodopsin-2 from *Chlamydomonas reinhardtii* (*Cr*ChR2).^5^ Ultrafast pump-probe experiments^6^ provided evidence for retinal isomerization and formation of the first photoproduct to take place with a time constant τ of 400 fs. Due to the fast deactivation of the excited state, the impact of retinal isomerization on the protein surrounding was observed with a time constant of 0.5 ps by Vis-pump/mid-IR probe spectroscopy.^7^

Much less is known about the photoreaction of the other light-activated cation channel of *C. reinhardtii*, *Cr*ChR1, due to the difficulties in overexpression. However, channelrhodopsin-1 from related *Chlamydomonas augustae* (*Ca*ChR1) achieves high expression yields in the yeast *Pichia pastoris*.^8,9^ Interestingly, *Ca*ChR1 comes with two distinct advantages for optogenetic application. It has a slower inactivation under sustained illumination than *Cr*ChR1 and a red-shifted absorption maximum as compared to *Cr*ChR2. Thus, *Ca*ChR1 can be activated with the light of longer wavelength, which is able to penetrate deeper into biological tissue.^8^

Like in *Cr*ChR2, the ground state of *Ca*ChR1 exhibits a heterologous retinal isomer composition. Retinal extraction and analysis of the isomers by high performance liquid chromatography reveals a 70:30 ratio of all-*trans* to 13-*cis* retinal. Resonance Raman experiments of the C=C stretching modes of the retinal embedded in the functional protein confirmed that mainly all-*trans* and to a minor amount 13-*cis* retinal exists.^9^ Recently, the heterogeneity of the ground state was again verified by UV/Vis absorption experiments with femtosecond time resolution which exhibit different photoreaction dynamics of *Ca*ChR1 on varying the excitation wavelength.^10^ These experiments revealed an ultrafast isomerization of the all-*trans* retinal to a hot and spectrally broad P_1_ photoproduct with a time constant of (100 ± 50) fs, followed by the photoproduct relaxation with time constants of (500 ± 100) fs and (5 ± 1) ps.^10^ UV/Vis absorption experiments with nanosecond time resolution showed that the appearance of a red-shifted intermediate P_1_ absorbing at around 560 nm arises, followed by the rise of a biphasic P_2_ intermediate matching the time for ion conduction of the channel.^11^ After the decay of the P_2_ intermediate, only faint traces of a red-shifted intermediate (P_3_-like) and a P_4_ intermediate have been detected.^11^

In this study, we focus on identification of the retinal all-*trans* photoreaction by vibrational spectroscopy. Time-resolved vibrational spectroscopy proved to be a very reliable method for characterization of photoisomerization dynamics.^12,13^ In particular, vibrational modes of retinal chromophores in photoreceptors are well studied. Vibrational marker bands for the all-*trans*, 15-*anti* retinal around 1163 cm^−1^, 1200 cm^−1^, and ∼1240 cm^−1^ were assigned to mixed C-C stretching modes of the chromophore. The vibration at ∼1240 cm^−1^ was assigned to a vibration with significant C_12_-C_13_ stretching mode character, while the vibration at ∼1200 cm^−1^ was assigned to a mode with significant C_14_-C_15_ stretching character.^14^ In *Ca*ChR1, these modes were observed in FTIR-difference spectra at 1163 cm^−1^, 1205 cm^−1^, and 1240 cm^−1^.^15^ Upon photoisomerization, the expected photoproduct has a 13-*cis*, 15-*anti* retinal conformation. A specific vibrational marker band was reported at about 1195 cm^−1^, assigned to a vibration with a significant C_14_-C_15_ stretching component.^16^ This marker band for a 13-*cis* photoproduct was also observed in *Ca*ChR1 by FTIR-difference spectroscopy and, very recently, by impulsive vibrational spectroscopy,^17^ confirming all-*trans* to 13-*cis* isomerization.^15^ Here, we focused on the time-resolved observation of the all-*trans* marker bands at 1200 cm^−1^ and 1240 cm^−1^, as well as on the 13-*cis*, 15-*anti* marker band at ∼1195 cm^−1^.

## II. RESULTS AND DISCUSSION

**FIG. 1.**
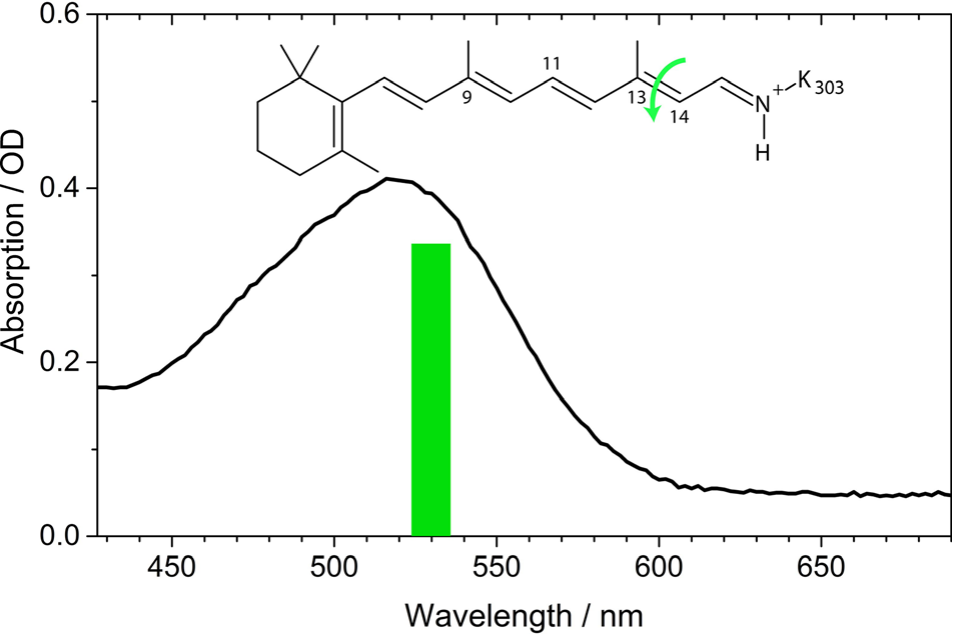
Absorption spectrum of *Ca*ChR1. The green bar shows the excitation wavelength. Inset: Retinal all-*trans*, 15-*anti* configuration with protonated Schiff base. Green arrow indicates the photoisomerization.

In Fig. 1, the visible absorption spectrum of *Ca*ChR1 is plotted. We excited the sample at ∼530 nm, where the absorption spectrum is dominated by *Ca*ChR1 with retinal all-*trans* configuration.

For tracking the retinal photoisomerization from all-*trans* to 13-*cis* configuration around the C_13_=C_14_ double bond, we applied angle-balanced polarization-resolved femtosecond visible (VIS) pump–IR probe spectroscopy^18^ to determine the vibrational dynamics on *Ca*ChR1 in H_2_O in the vibrational fingerprint region from 1174 cm^−1^ to 1257 cm^−1^ with a high spectral resolution of 1.5 cm^−1^. The spectral range around 1200 cm^−1^ exhibits well-characterized vibrational marker bands for retinal all-*trans* conformation, and retinal 13-*cis* conformation. These vibrational modes are dominated by the C_14_-C_15_ stretching vibration of the retinal chromophore, the position where photoisomerization is supposed to induce strongest alterations. Since electronic spectra of stimulated emission, electronic excited state, product, and ground-state absorption overlap considerably, the vibrational marker band at 1190 cm^−1^ is a very suitable tool for 13-*cis* photoproduct identification.

**FIG. 2.**
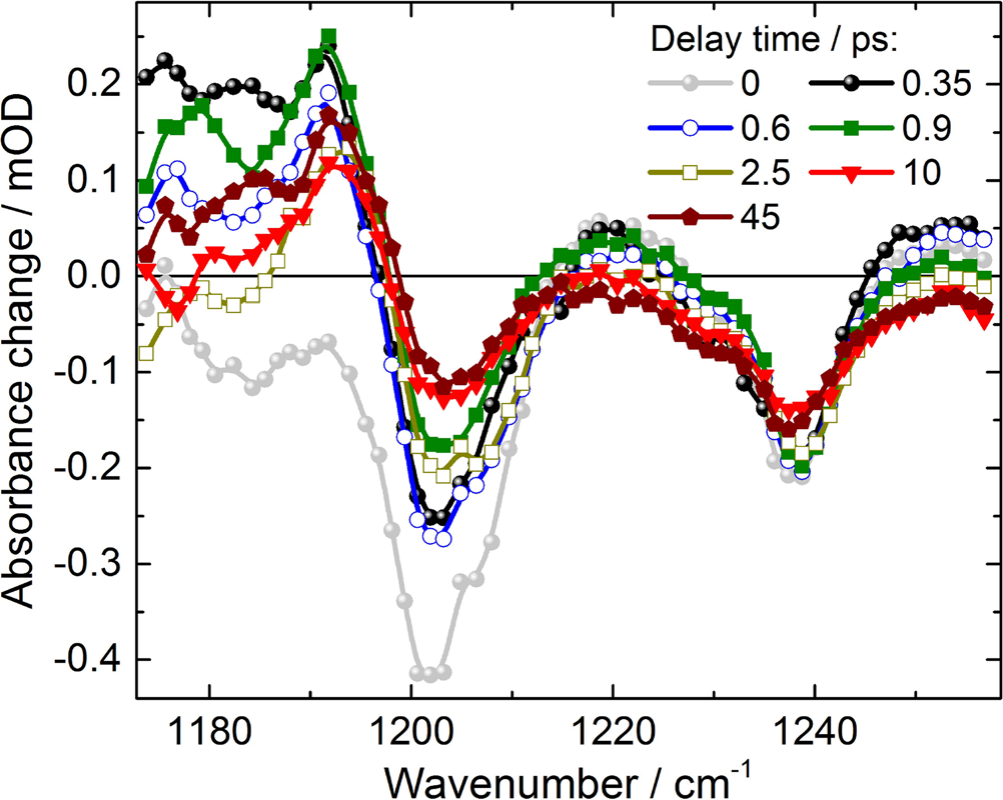
Absorbance difference spectra of *Ca*ChR1 upon excitation at 530 nm at specific pump-probe delay times. Negative signals are bleaching signals; positive signals show vibrational absorption of hot ground-states, excited states, or product bands.

**FIG. 3.**
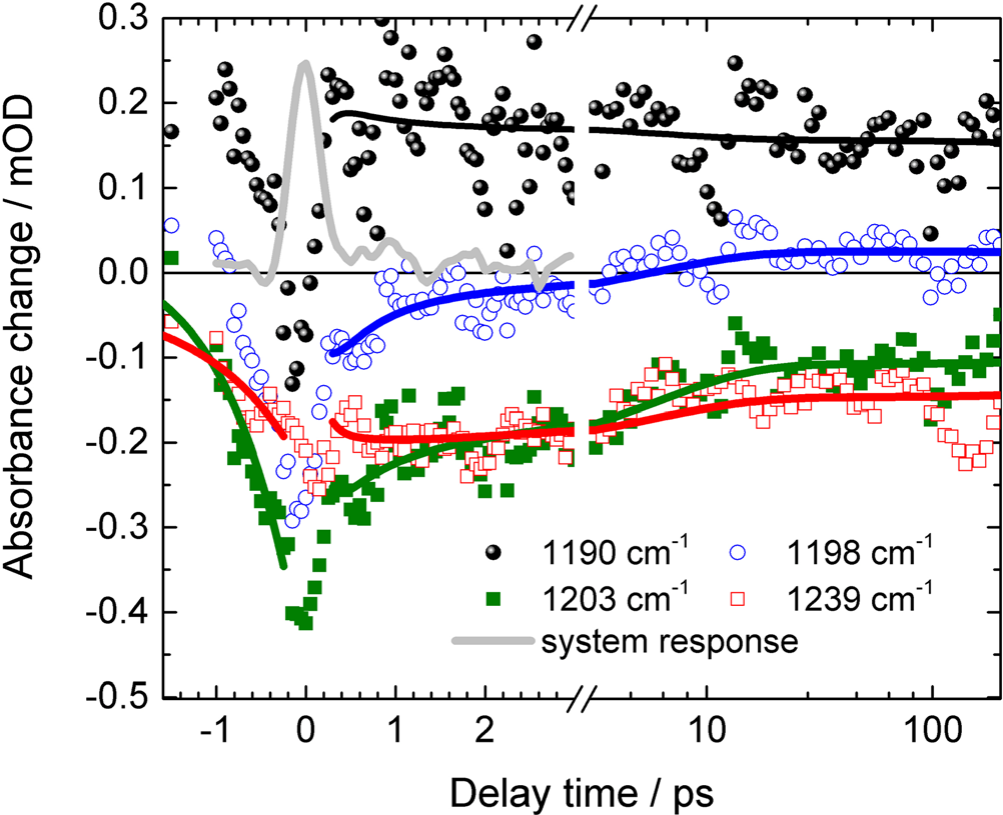
Transients of *Ca*ChR1 upon excitation at 530 nm for selected wavenumbers. Positive delay times: Solid lines represent simulations with a sum of three exponentials; negative delay times: Solid lines represent PFID signals at 1203 cm^−1^ (green line) with a time constant of (0.80 ± 0.08) ps, and at 1239 cm^−1^ (red line) with a time constant of (1 ± 0.2) ps.

Upon photoexcitation at 530 nm, spectral changes in the fingerprint region are presented in Fig. 2 for selected pump–probe delay times. An instantaneous strong negative signal is observed at 1203 cm^−1^, reflecting the ground-state bleaching of the C_14_-C_15_ stretching vibration of retinal in the all-*trans* conformation. A strong positive signal is visible at 1190 cm^−1^, representing the C_14_-C_15_ stretching vibration of the 13-*cis* conformation. At time zero, this positive signal is absent, but at a delay time of 0.35 ps the signal has reached its maximum. This points to a very fast formation of the retinal 13-*cis* photoproduct, which is faster than 0.3 ps. Upon excitation, strong mixing of the C=C double and C-C single bond vibrations takes place in the electronic excited state. Thus, we observe no strong positive signal from retinal excited state absorption in the investigated spectral region. Another significant negative signal is visible at 1239 cm^−1^, displaying the bleaching band of C_12_-C_13_ stretching vibration in the retinal all-*trans* conformation. This band is spectrally shifted in the 13-*cis* conformation, and has negligible spectral overlap with strong positive absorption bands. Hence, we can use the bleaching recovery of this band to estimate the forward quantum yield of the photoreaction. The high spectral resolution of 1.5 cm^−1^ allows for identification of spectral substructures. A closer inspection of the bleaching band around 1239 cm^−1^ shows that a negative shoulder in the bleaching band at around 1230 cm^−1^ exists. Furthermore, we observe a broad positive feature from 1215 cm^−1^ to 1257 cm^−1^ at early delay times, which decays within a picosecond completely. In contrast, a remaining positive band at 1220 cm^−1^ is observed in the FTIR-difference spectra at 80 K.^19^ This could point to the trapping of a transient intermediate state at low temperatures which relaxes back to the parent all-*trans* ground state on a picosecond time scale at room temperature. The transients at selected spectral positions plotted in Fig. 3 provide information on the photoreaction dynamics. At negative delay times, i.e., when the probe pulse arrives at the sample before the pump pulse, we observe signals from the perturbed free induction decay (PFID).^20^ This results in an exponential increase at spectral positions of the strong bleaching bands at 1239 cm^−1^ and 1203 cm^−1^ (plotted as red and green lines in Fig. 3), reflecting the dephasing of these vibrations. The exponential rise of the PFID signal at 1239 cm^−1^ corresponds to a line width (FWHM) of (10 ± 2) cm^−1^,^20^ matching the line width of the bleaching signal at 45 ps in Fig. 2, after completion of cooling processes. This supports the absence of a positive signal superimposed at 1239 cm^−1^ for long delay times. At time zero, the pump pulse arrives and populates excited states. Within the system response of 0.3 ps (grey line in Fig. 3) the all-*trans* bleaching signal at 1203 cm^−1^ (green line) appears and decays on a sub-picosecond to picosecond time scale. This bleaching recovery can reflect either repopulation of the ground state, or a blue-shift of the adjacent positive C_14_-C_15_ stretching vibration of the 13-*cis* conformation around 1190 cm^−1^. The latter is supported by narrowing of the spectral width of the C_14_-C_15_ stretching vibration around 1180 cm^−1^, and the blue-shift of the zero-crossing around 1197 cm^−1^ for increasing delay times in Fig. 2.

The transient of the C_14_-C_15_ stretching vibration of the 13-*cis* conformation (ν(C_14_-C_15_)^13cis^) at ∼1190 cm^−1^ (black circles in Fig. 3) displays a positive signal, which rises within the system response of 0.3 ps. Since the band at 1190 cm^−1^ is a marker band for 13-*cis* conformation, we can conclude that photoproduct formation due to all-*trans* isomerization is finished after 0.3 ps. This is in line with recent studies on electronic transitions.^10^ The transient stays nearly constant in amplitude within the observed time window. All data are well-simulated by the sum of three exponentials.

**FIG. 4.**
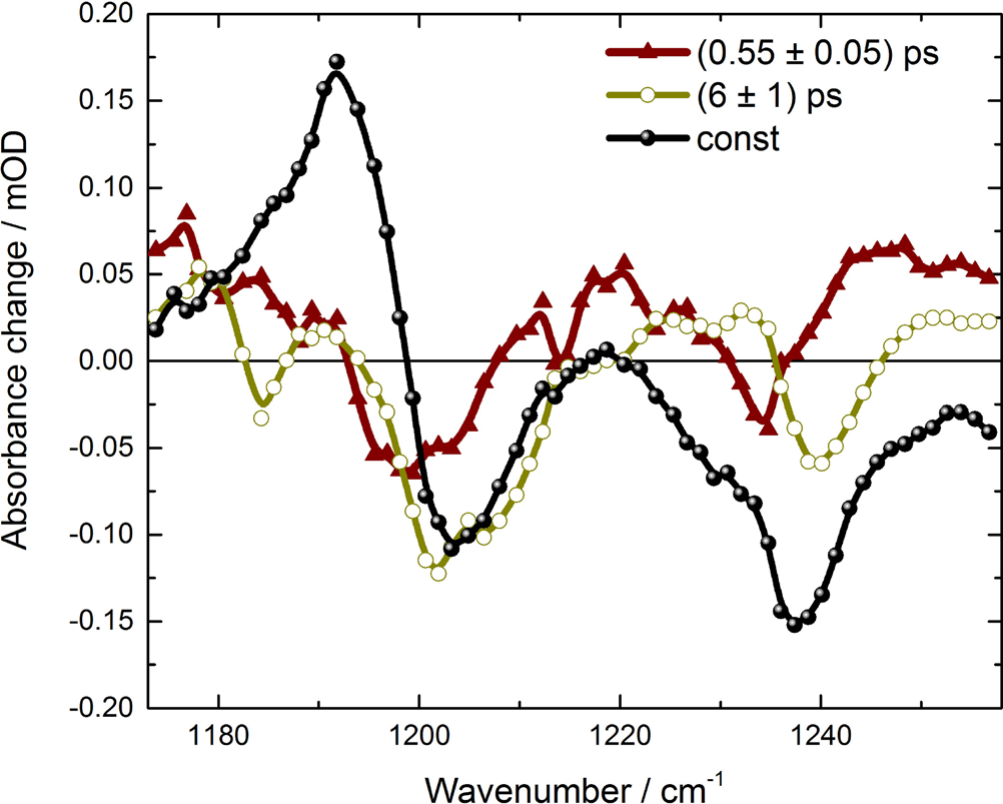
Decay associated spectra of *Ca*ChR1. The constant DAS_τ3_ (black) represents the difference spectra on a time scale of hundreds of ps. The fast DAS_τ1_ (brown) shows dispersive features of vibrational cooling. DAS_τ2_ (dark yellow) exhibits dispersive features and features of the bleaching bands.

We found decay constants of τ_1_ = (0.55 ± 0.05) ps, τ_2_ = (6 ± 1) ps, and a decay constant τ_3_ much longer than our observation time window. We assign τ_3_ to the remaining and constant signal in our time window of 200 ps. The simulated curves are presented in Fig. 3 as solid lines, and at spectral positions of 1203 cm^−1^ and 1239 cm^−1^ simulations of the PFID are also displayed. The transient of the ν(C_14_-C_15_)^trans^ bleaching band at 1203 cm^−1^ (green line, Fig. 3) exhibits a significant decay with 0.55 ps and 6 ps. In contrast, the transient of the ν(C_12_-C_13_)^trans^ bleaching band at 1239 cm^−1^ shows only a small amplitude changes with a decay constant of 6 ps. The decay associated spectra (DAS) of the decay constants τ_1_ (DAS_τ1_), τ_2_ (DAS_τ2_), and τ_3_ (DAS_τ3_) are plotted in Fig. 4.

The fast component DAS_τ1_ exhibits a broad positive signal from 1173 cm^−1^ to 1193 cm^−1^, and a negative signal with similar strength from 1193 cm^−1^ to 1208 cm^−1^. The zero-crossing is exactly at the maximum of the ν(C_14_-C_15_)^13cis^ absorption, indicating vibrational cooling of the 13-*cis* product band. Moreover, a broad positive signal from 1208 cm^−1^ to 1258 cm^−1^ is visible, with a negative peak around 1234 cm^−1^. This feature could be caused by cooling of a vibration absorbing at higher wavenumbers than 1258 cm^−1^. We assign the 0.55 ps component solely to the cooling processes. The slower component DAS_τ2_ has also a positive component below 1193 cm^−1^, and negative contributions from 1193 cm^−1^ up to 1220 cm^−1^. In contrast to the fast component DAS_τ1_, the negative signal has more amplitude than the positive one. For pure vibrational cooling processes, one would expect a stronger positive signal compared to the negative signal of the same vibration, since the oscillator strength is typically increased by vibrational excitation. This could point to the cooling processes, overlapped by ground-state recovery. This is supported by the positive/negative feature at 1228 cm^−1^ (+)/1239 cm^−1^ (−) in DAS_τ2_. Again, a positive signal is observed at higher wavenumbers (around 1250 cm^−1^). Moreover, the DAS_τ2_ has negligible contributions at 1193 cm^−1^, but strong contributions at 1203 cm^−1^ indicating no increase of the 13-*cis* photoproduct, but recovery of the all-*trans* bleaching band with a decay time of 6 ps. This supports the assignment of back-reaction processes with a time constant of 6 ps. The constant signal DAS_τ3_ shows the clear positive/negative signature at 1192 cm^−1^ (+)/1204 cm^−1^ (−) of all-*trans* to 13-*cis* photoisomerization with amplitude ratio of 3:2, similar to those observed in light-induced FTIR-difference spectra of *Ca*ChR1 at cryogenic temperature.^19^

**FIG. 5.**
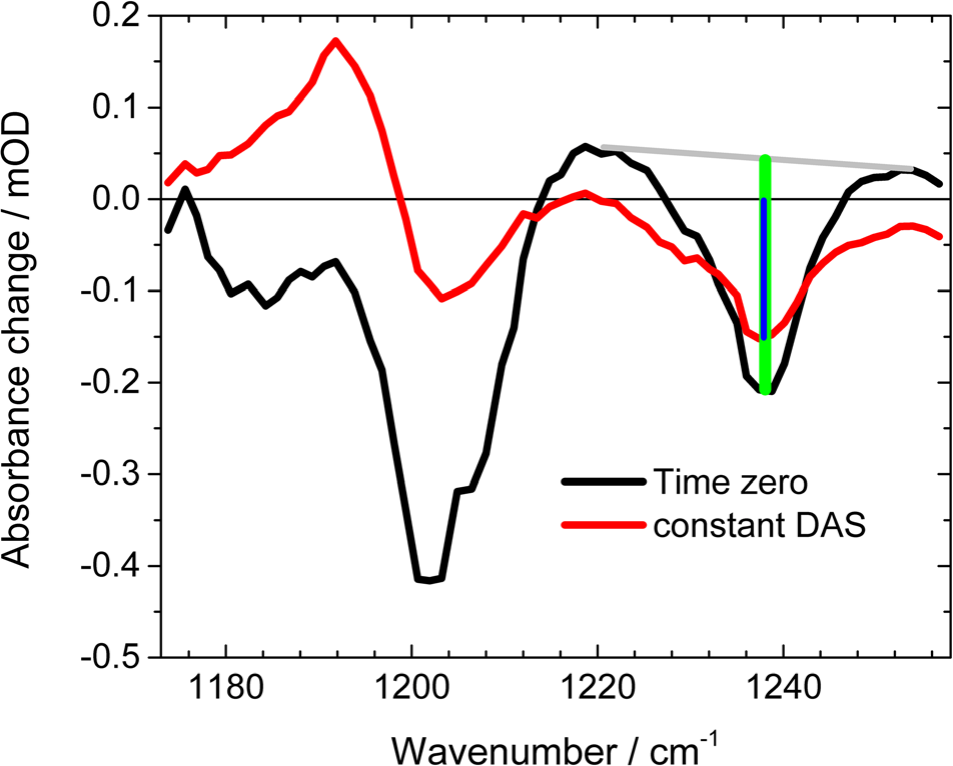
Absorbance difference spectra at time zero (black line), and at long delay times represented by DAS_τ3_ (red line). At 1239 cm^−1^ the amplitudes are taken at time zero and for long delay times, represented by the green and blue bars, respectively. Grey line: Baseline for subtraction of the positive broad background at time zero.

Since the ν(C_14_-C_15_)^trans^ bleaching band at 1203 cm^−1^ is strongly masked by the ν(C_14_-C_15_)^13cis^ absorption, we analyzed the ν(C_12_-C_13_)^trans^ bleaching band at 1239 cm^−1^ to estimate the quantum yield of the forward photoisomerization reaction. In Fig. 5, we present the different absorption spectrum at delay time zero (black line) together with the constant component DAS_τ3_ from the simulations. At 1239 cm^−1^, we see negligible contributions of non-linear spectral features, but a broad positive background at delay time zero. By subtracting the background approximated by a straight line (grey line in Fig. 5), the bleaching signal strength is calculated at time zero (green line in Fig. 5). The constant DAS in Fig. 5 (red line) shows the pure bleaching signal at 1239 cm^−1^ without overlapping the positive contributions. The bleaching signal strength after the photoreaction is calculated and presented in Fig. 5 (blue line). The ratio of the two bleaching signals reveals the proportion of *Ca*ChR1 not reacting back to the all-*trans* ground state, but undergo a forward reaction. Thus, the forward reaction quantum yield can be determined from our data to (0.60 ± 0.06).

Closer inspection of the spectral shape of the bleaching band at 1239 cm^−1^ displays a shoulder at 1230 cm^−1^. This shoulder is visible in all difference spectra (Fig. 2) indicating an additional bleaching band. Whether this bleaching band reflects retinal ground state heterogeneity in all-*trans*, 15-*anti* conformation, i.e., due to different hydrogen bonding, or is caused by a sub-population of 13-*cis*, 15-*syn* retinal^10,21^ of ground-state *Ca*ChR1 will be investigated in future studies.

## III. CONCLUSION

We present the first femtosecond time-resolved IR study of the photoisomerization of channelrhodopsin-1 from *Chlamydomonas augustae* (*Ca*ChR1) in the vibrational fingerprint region of the C-C stretching vibrations. The vibrational dynamics of the retinal chromophore isomerization from all-*trans* to 13-*cis* was investigated by polarization-resolved VIS pump mid-IR probe spectroscopy at a high time resolution (about 300 fs). After photoexcitation at 530 nm, the transient infrared absorption was probed in a spectral region with dominant C-C stretching mode absorption. The photoproduct C_14_-C_15_ vibrational marker mode at 1190 cm^−1^ that is indicative for a 13-*cis*, 15-*anti* configuration of the chromophore rises within the time resolution. Investigations in the visible spectral range reported photoisomerization time constants of 100 fs.^10^ This is in line with our observations that provide direct evidence for the isomerization taking place faster than 0.3 ps, faster than in bacteriorhodopsin^13^ or in channelrodopsin-2^7^ from *Chlamydomonas reinhardtii* (*Cr*ChR2). Vibrational dynamics show additional time constants of (0.55 ± 0.05) ps and (6 ± 1) ps, identical to those observed in ultrafast VIS pump supercontinuum probe experiments.^10^ We assigned the 0.55 ps time constant predominantly to vibrational cooling, while the longer time constant of 6 ps probably also consists of a back-reaction pathway. We estimated the photoisomerization reaction yield by the bleaching signal of the C_12_-C_13_ stretching band at 1239 cm^−1^ to (60 ± 6)%, very similar to other rhodopsins. Our high spectral resolution of 1.5 cm^−1^ allows for identification of an additional bleaching component at 1230 cm^−1^. This finding strongly supports ground state heterogeneity of the retinal chromophore. Further studies should be performed to assign this bleaching band to either heterogeneity of the all-*trans*, 15-*anti* retinal or to a 13-*cis*, 15-*syn* (dark-adapted) retinal conformation. Our study clearly demonstrates various different photoreaction processes in retinal photoreceptors. *Ca*ChR1shows a significantly faster isomerization dynamics as *Cr*ChR2 at a high yield. Further studies will be performed to identify the molecular origin of these differences, in order to truly understand the optimization of photoreactions in photoreceptors.

## IV. METHODS

*Ca*ChR1 was prepared as described previously.^9,22^ Briefly, the truncated *Ca*ChR1 gene (1-352 aa) was fused with a 10xHis-tag (GeneArt, Life Technologies) and was heterologously expressed in *Pichia pastoris* yeast cells. The solubilized protein was purified on a Ni-NTA column (Macherey-Nagel, Germany) and concentrated to 46 mg/ml in a buffer containing 20 mM Hepes, 100 mM NaCl, 0.05% dodecyl maltoside at pH 7.4. Two times 150 *μ*l of the *Ca*ChR1 solution was placed between two CaF_2_ windows. The spectral line-width of the femtosecond excitation pulses is sketched with the absorption spectrum of *Ca*ChR1 in Figure 1.

Femtosecond laser pulses were generated starting from a fundamental femtosecond laser pulse delivered by a 1 kHz Ti:Sa laser system (Coherent Legend USP, 80 fs pulses at 800 nm). The fundamental beam was split into two parts for pump and probe pulse generation. The pump pulses were generated in a non-collinear optical parametric amplifier (NOPA). A sapphire white light supercontinuum was used as seed, amplified in a β-barium borate (BBO) crystal by frequency doubled pulses at 400 nm. We selected energies to excite the sample of about 0.4–0.5 *μ*J per pulse with a pump focus diameter of about 300 *μ*m.

Angle balanced femtosecond polarization resolved VIS pump–IR probe measurements were applied as described elsewhere.^18^ In short, the mid-IR probe beam is generated by a difference-frequency mixing of near-infrared signal and idler pulses generated by 800 nm fs pulses in a BBO crystal. Two reflections of the fs mid-IR pulse are taken as probe beams with different polarizations used at the same time in the same sample volume to detect absorbance changes. The system response was about 300 fs (shown in Fig. 3, grey line). We measured the system response in a thin Ge plate in identical sample holders, as were used for the experiments on *Ca*ChR1. Absorbance changes with mid-IR polarizations parallel (A_pa_) and perpendicular (A_pe_) to the VIS pump beam polarization were detected. Isotropic absorbance changes (A_iso_) were calculated by A_iso_ = (A_pa_ + 2 A_pe_)/3. Here, we presented only isotropic data.
